# Identification of absorbed constituents and evaluation of the pharmacokinetics of main compounds after oral administration of yindanxinnaotong by UPLC-Q-TOF-MS and UPLC-QqQ-MS†

**DOI:** 10.1039/c7ra12659j

**Published:** 2018-04-26

**Authors:** Leilei Gong, Haiyu Xu, Huijun Yuan, Lan Wang, Xiaojie Yin, Moqi Fan, Long Cheng, Xiaojing Ma, Rixin Liang, Hongjun Yang

**Affiliations:** Institute of Chinese Materia Medica, China Academy of Chinese Medical Science No. 16 Nanxiaojie, Dongzhimennei Ave. Beijing 100700 P. R. China liangrixin2009@sina.com Hongjun0420@sina.com; School of Traditional Chinese Medicine, Capital Medical University Beijing 100069 P. R. China; The first clinical medical college of Beijing University of Chinese Medicine Beijing 100700 P. R. China; Key Laboratory of Bioactive Substance and Resource Utilisation of the Chinese Herbal Medicine Ministry of Education, Institute of Medicinal Plant Development, Chinese Academy of Medical Sciences, Peking Union Medical College Beijing 100193 P. R. China

## Abstract

Yindanxinnaotong capsule (YDXNT), a traditional Chinese formula, has been used to treat cardio-cerebrovascular diseases for several decades. Previous research has focused on evaluating the pharmacological properties and main compounds of YDXNT *in vitro* and *in vivo*. However, the multiple bioactive compounds *in vivo* remain poorly understood. In the present research, an integrative strategy using UPLC-Q-TOF-MS combined with UPLC-QqQ-MS was employed to detect the absorbed constituents and investigate the pharmacokinetics of main compounds in the plasma after oral administration of YDXNT. UPLC-Q-TOF-MS was developed to detect the absorbed constituents and their metabolites in the plasma after oral administration in rats. A total of 52 constituents, including 44 prototype compounds and 8 metabolites, were identified or tentatively characterized. Then, nine main compounds (quercetin, isorhamnetin, kaempferol, ginkgolide A, ginkgolide B, ginkgolide C, bilobalide, tanshinone IIA, and salvianolic acid B) were chosen to further investigate the pharmacokinetic behavior of YDXNT using UPLC-QqQ-MS. The concentration of nine main constituents were in the range of 27.85–76.54 ng mL^−1^. This research provides a systematic approach for rapid qualitative analysis of absorbed constituents and for evaluating the pharmacokinetics of the main ingredients of YDXNT following its oral administration. More importantly, this work provides key information on the identification of bioactive compounds and the clarification of their action mechanisms, as well as on the pharmacological actions of YDXNT.

## Abstract 2

Yindanxinnaotong capsule (YDXNT), a traditional Chinese formula, has been used to treat cardio-cerebrovascular diseases for several decades.

## Graphical abstract

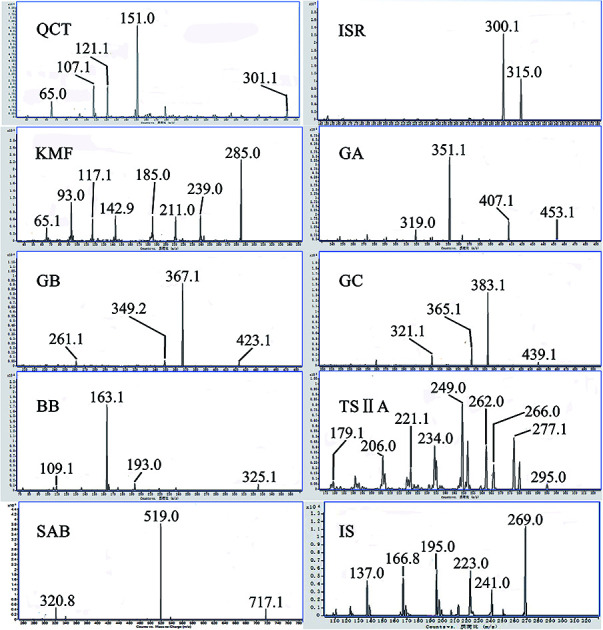

## 1. Introduction

Traditional Chinese medicine (TCM), one of the oldest phytomedicine systems in health care, has been used in Asia, such as in China, Korea, and Japan, for thousands of years.^1–4^ The herbs used in TCM usually have complex formulas and function mainly *via* their multiple constituents, targets and modes of action (3M) in a network-based and holistic manner.^5^ Thus, the therapeutic effects of TCM formulae rely on the joint effect of multiple ingredients. Most TCM formulae are taken orally, and only ingredients absorbed into the blood can exert their bioactivities.^6^ Therefore, it is necessary to trace the compounds in a TCM prescription *in vivo* and evaluate their pharmacokinetics to provide more in-depth insight into the main active components and therapeutic mechanisms of TCM formulae. In recent years, an integrative strategy using ultra-high-performance liquid chromatography coupled with quadrupole time-of-flight mass spectrometry (UPLC-Q-TOF-MS) combined with ultra-high-performance liquid chromatography with triple quadrupole mass spectrometry (UPLC-QqQ-MS) has been widely used to identify the absorbed constituents and determine main compounds in complex matrices.^7–11^ Hence, they are valuable analytical techniques for identifying key compounds and evaluating the pharmacokinetics of TCM formulae *in vivo*.

Yindanxinnaotong capsule (YDXNT), a classic TCM formula, comprises eight herbs: *Ginkgo biloba* leaf (yinxingye, YXY), *Salvia miltiorrhizae* (danshen, DS1), *Gynostemma gynostemmatis* (jiaogulan, JGL), *Erigerontis herba* (dengzhanxixin, DZXX), *Allii sativi bulbus* (dasuan, DS2), *Radix*/*rhizoma notoginseng* (sanqi, SQ), *Crataegi fructus* (Shanzha, SZ), and *Borneolum* (tianranbingpian, TRBP). In China, YDXNT capsule is officially listed in the Chinese Pharmacopoeia 2015 as a prescription for patients suffering from angina pectoris in CHD, hyperlipidemia, stroke, and atherosclerosis.^12^ YDXNT, which has been used to treat cardio-cerebrovascular diseases for many years,^13–15^ is one of the stasis-eliminating formulated products listed in China's National Basic Medical Insurance Drugs Catalogue (http://www.nhfpc.gov.cn/, 2013 edition).

Our previous research focused on the chemical compounds, quality control, and *in vitro* and vivo pharmacological properties of YDXNT. In studying the chemical compounds of YDXNT, 70 chemical constituents, including 23 flavonoids, 4 ginkgolides, 15 phenolic compounds, 18 diterpenoid tanshinones, and 10 ginsenosides, were tentatively identified using various detection approaches. Furthermore, fingerprint analysis was established to evaluate the uniform quality of YDXNT soft capsules.^16^ In *in vivo* experiments, taking YDXNT and swimming may prevent atherosclerosis through a synergistic effect between YDXNT and swimming leading to improved blood circulation, hemorheological parameters, blood lipid levels and vascular endothelium in rats.^17^ In an isolated heart experiment, YDXNT and its main compounds elevated heart function, coronary flow and SOD levels and decreased levels of MDA and inflammatory factors.^18^ Ultimately, our research demonstrated that YDXNT and its major constituents relieve atherosclerosis by regulating lipids, reducing lipid particle deposition in the endothelial layer of arteries, enhancing antioxidant power, and repressing inflammatory activity by inhibiting the nuclear factor-kappa B signaling pathway both *in vivo* and vitro.^19^ Previous work focus on the pharmacokinetics of main constituents in YXY, DS1, SQ, SZ, and DZXX.^20–24^ However, until now, little has been known about how many constituents are absorbed into the blood after oral administration of YDXNT or about the final fate of the formula *in vivo*. This presents a huge obstacle to understanding the pharmacological mechanisms of YDXNT. Thus, the present research was designed to determine the absorbed compounds and metabolites in biological samples from rats and investigate the pharmacokinetics of the main abundant active components.

In this research, UPLC-Q-TOF-MS was used to analyze and identify the absorbed ingredients and possible metabolites in rat plasma after oral administration of YDXNT due to its high chromatographic resolution, high sensitivity, short analysis times, good separation, and highly accurate mass values.^25–27^ In addition, UPLC-QqQ-MS was used for simultaneous quantitation of the main components quercetin (QCT), kaempferol (KMF), isorhamnetin (ISR), ginkgolide A (GA), ginkgolide B (GB), ginkgolide C (GC), bilobalide (BB), tanshinone IIA (TSIIA), and salvianolic acid B (SAB) in rats, with baicalein used as the internal standard (IS).^28,29^ These compounds exert anti-atherosclerotic and endothelium-independent vasodilator effects, prevent inflammatory damage and have a protective effect on nitric oxide, antioxidant action, and anti-vascular inflammation.^26–29^ The pharmacokinetics of QCT, KMF, ISR, GA, GB, GC, BB, TSIIA, and SAB were investigated in rats after oral administration of YDXNT, and the pharmacokinetics properties of this formula were further speculated. This research represents the first detailed investigation into the absorbed constituents, metabolites, and pharmacokinetics of YDXNT. It may provide valuable information for better understanding the pharmacological mechanisms and clinical applications of YDXNT.

## 2. Experiments

### 2.1 Chemicals and reagents

YDXNT soft capsules were provided by Guizhou Bailing Group Pharmaceutical Co., Ltd. Standards of rutin and cryptotanshinone were purchased from Chengdu Chroma-Biotechnology Co., Ltd. (Sichuan, China). Kaempferol, quercetin, tanshinone IIA, salvianolic acid B, ginkgolide A, ginkgolide B, bilobalide, and baicalein were purchased from Chengdu Herb Purify Co., Ltd. (Sichuan, China). Isorhamnetin, ginkgolide C, ginsenoside Rg1, ginsenoside Re, ginsenoside Rd, and notoginsenoside R1 were purchased from the National Institutes for Food and Drug Control (Beijing, China), Ltd., and HPLC-grade acetonitrile and methanol were obtained from Thermo Fisher Scientific Inc. (Shanghai, China). Formic acid was purchased from Sinopharm Chemical Reagent Co., Ltd. (Shanghai, China), and the water used in the experiments was purified with a Milli-Q system (Sartorius Arium® Pro, Germany).

### 2.2 Preparation of YDXNT sample

The samples were first processed by completely removing the outer capsule layer. Then, a 48.0 g sample was precisely weighed and soaked with 100 mL de-ionized water. The sample was ultrasonically dissolved for 30 min and then stored at −4 °C until it was orally administered to rats.

### 2.3 Animal experiments

All animal procedures were performed in accordance with the Guidelines for Care and Use of Laboratory Animals of Center for Laboratory Animal Care, China Academy of Chinese Medical Science and approved by the Animal Ethics Committee of Institute of Chinese Material Medica, China Academy of Chinese Medical Science.

Twenty-four male Sprague-Dawley rats (250 ± 30 g) were obtained from the Laboratory Animal Center of the Academy of Military Medical Sciences (Beijing, China) and divided into four groups (six rats each). The rats were housed and fed at a temperature of 25 ± 2 °C and a relative humidity of 50% for three days in the breeding room. Standard chow and deionized water were provided prior to the oral treatment. All rats were fasted for 12 h with free access to water before the experiment. YDXNT was administered orally to rats at a dose of 4.8 g kg^−1^ d^−1^, and blood was collected from the abdominal aorta after 20% urethane anesthesia and then centrifuged at 3000 rpm for 10 min at 4 °C to obtain the plasma. All samples were stored at −20 °C until analysis. Blank plasma samples were prepared in the same manner.

### 2.4 Serum sample preparation

Six milliliters of 75% methanol was added to two milliliters of prepared plasma and then vortexed for 60 s. The mixture was centrifuged at 4000 rpm for 10 min at 4 °C. The supernatant was transferred to another EP tube and dried using a pressure blowing concentrator. The residue was redissolved with 200 μL of 75% methanol, vortexed for 60 s, and then centrifuged at 13 000 rpm for 10 min. To an aliquot of 200 μL plasma, 120 μL of 0.2 mol L^−1^ acetic acid–sodium acetate buffer solution (pH = 4.6), 100 μL of the IS baicalein and 80 μL of 980 U mL^−1^ β-glucuronidase and sulfatase was added and then vortexed for 1 min. The mixture was hydrolyzed in a water bath at 37 °C for 30 min, and 600 μL acetone was added to stop the hydrolysis. The sample was cooled, followed by vortexing for 2 min and then centrifugation at 13 000 rpm for 10 min. The supernatant fluid was evaporated to dryness under a flow of nitrogen gas.

### 2.5 Preparation of standard, calibration, and quality control solutions

Stock solutions of QCT, ISR, KMF, GA, GB, GC, BB, TSIIA, SAB and IS were separately prepared by dissolving the compounds in methanol at a concentration of 200 ng mL^−1^; all solutions were stored at 4 °C. Working solutions for calibration and QC samples were prepared by adding the diluted stock solutions to blank rat plasma. Calibration standards of plasma-derived working solutions with QCT, ISR, KMF, GA, GB, GC, BB, and TSIIA were all prepared with final concentrations ranging from 1–100 ng mL^−1^. The SAB solution was prepared with final concentrations ranging from 5–500 ng mL^−1^. The IS solution was prepared at a final concentration of 100 ng mL^−1^ in methanol. For method validation, QC plasma samples of QCT, ISR, KMF, GA, GB, GC, BB, and TSIIA were all prepared separately at three concentrations (1, 10 and 100 ng mL^−1^). The QC plasma sample of SAB was prepared at three concentrations (5, 50, and 500 ng mL^−1^).

### 2.6 UPLC-Q-TOF-MS for qualitative analysis

UPLC data were produced using the Waters Synapt G2-S UPLC system (Waters, Milford, USA) equipped with a quaternary pump system, online degasser, autosampler, thermostatically controlled column compartment, and diode array detector. The system was controlled with Masslynx V4.1 software. The chromatographic column ACQUITY BEH C18 (100 × 2.1 mm, 1.7 μm) equipped with VanGuard™ BEHC18 1.7 μm was used. The column was eluted with a gradient of A (0.1% formic acid in deionized water) and B (CAN) at a flow rate of 0.3 mL min^−1^ and a temperature of 30 °C: 0–2.5 min, 10–16% B; 2.5–6.5 min, 16–16% B; 6.5–9.5 min, 16–25% B; 9.5–11.0 min, 25–25% B; 11.0–12.0 min, 25–45% B; 12.0–15.0 min, 45–55% B; 15.0–18.0, 55–98% B; 18.0–21.0 min, 98–98% B; 21.0–25.0 min, 98–10% B. An injection volume of 10 μL was used for analysis.

For mass detection, mass data were recorded using the Waters Synapt G2-S Q-TOF Mass Spectrometer system equipped with an atmospheric pressure chemical ionization (APCI) electrospray ionization (ESI) interface (Waters, Milford, USA). The conditions of the TOF/MS were set as follows: capillary, 2.5 kV; source temperature, 120 °C; cone gas flow, 50 L h^−1^; nebulizer, 6 bar; desolvation temperature, 450 °C; sheath gas flow, 12 L min^−1^; nozzle voltage, 10–1000 V; The mass spectrometer was operated from 100 to 1700 *m*/*z*. Accurate masses and compositions of the precursor and fragment ions were calculated using MassLynx 4.1 software. The UPLC and Q-TOF-MS methods have been optimised (ESI 1†).

### 2.7 UPLC-QqQ-MS for quantitative analysis

Chromatographic analysis was performed using an Agilent 1290 Series UPLC from Agilent Technologies (Palo Alto, CA, USA), equipped with a quaternary pump an online degasser, auto-sampler and column oven. Chromatographic separation was performed on the Agilent ZORBAX SB-Aq C18 column (100 mm × 2.1 mm, 1.8 μm, Agilent, USA). The analytical column temperature was maintained at 30 °C and eluted with a mobile phase comprising 0.1% formic acid in water (A) and acetonitrile (B) using the following gradient program: 25–90% B from 0 to 1 min, 90% B from 1 to 3.5 min, and 90–25% B from 3.5 to 5 min at a flow rate of 0.3 mL min^−1^. The equilibration time was 5 min, and the injection volume of sample and reference constituents was 5 μL.

**Fig. 1:**
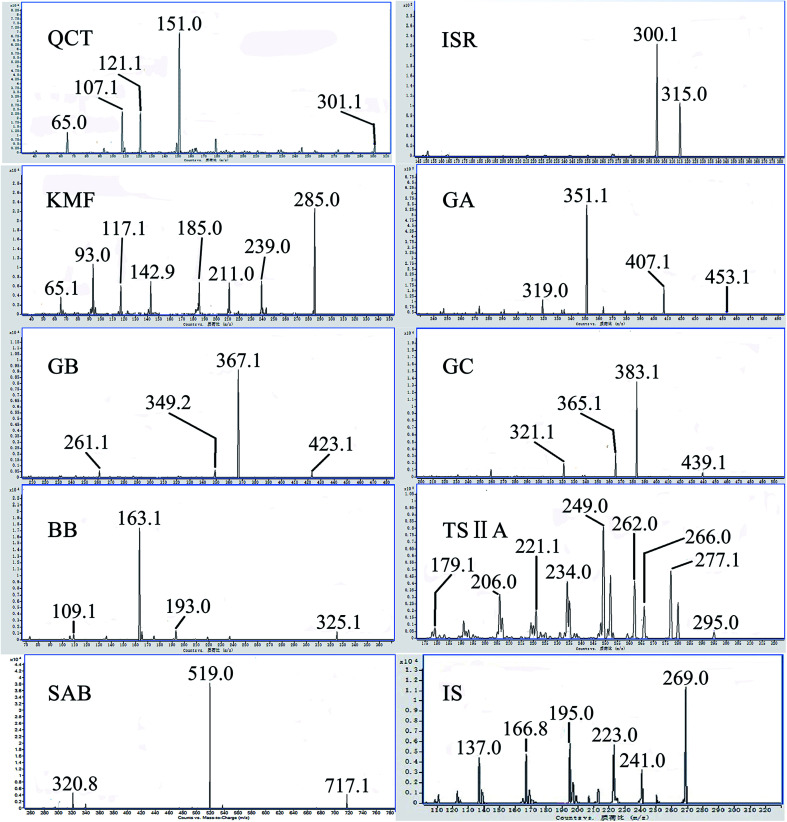
Product iron mass spectra of the analytes and internal standard (IS). QCT: quercetin; ISR: isorhamnetin; KMF: kaempferol; GA: ginkgolide A; GB: ginkgolide B; GC: ginkgolide C; BB: bilobalide; TSIIA: tanshinone IIA; SAB: salvianolic acid B; IS: baicalein.

Mass spectrometry was performed on the Agilent 6490 triple-quadrupole mass spectrometer (Palo Alto, CA, USA) equipped with an atmospheric pressure chemical ionization (APCI) interface. The mass spectrometer was set in negative ionization mode, with the capillary voltage set at 3500 V. The other parameters in the source were set as follows: desolvation gas flow, 15 L min^−1^; source temperature, 290 °C; N_2_ sheath gas temperature, 350 °C; nebulizer gas (N_2_) pressure, 50 psi; flow, 12 L min^−1^. The mass spectrometer scanned in multiple reaction monitoring (MRM) mode. The UPLC and QqQ-MS methods have been optimised (ESI 2†). Fig. 1 shows the MS/MS product ion spectra of the analytes and IS. The optimized extraction ion pairs and parameter values are shown in Table 1.

**Table:** MRM transitions and parameters for the detection of QCT, ISR, KMF, GA, GB, GC, BB, TSIIA, SAB and IS

Analyte	MRM transitions	Fragementor (V)	Collision energy (V)	Dwell time (ms)
QCT	301.1 → 151.0	350	26	20
ISR	315.0 → 300.1	350	22	20
KMF	285.0 → 142.9	350	35	20
GA	453.1 → 351.1	350	16	20
GB	423.1 → 367.1	350	15	20
GC	439.1 → 383.1	350	12	20
BB	325.1 → 163.1	350	16	20
TSIIA	295.0 → 249.0	350	26	20
SAB	717.1 → 519.0	350	38	20
IS	269.0 → 195.0	350	28	20

### 2.8 Method validation

Method validation was performed according to the guidelines for bioanalytical method validation published by the US FDA.^30^ The analytical method used in this research was validated to demonstrate its specificity, selectivity, linearity, lower limit of quantification (LLOQ), precision, accuracy, stability, extraction recovery rates and matrix effects of the samples (ESI 3†).

### 2.9 Pharmacokinetics study

Six male rats were used in the pharmacokinetics study. All the rats were orally administered 2.4 g kg^−1^ YXCNT suspended in an aqueous solution of 0.5% carboxymethyl cellulose sodium (QCT: 4.118 mg; KMF: 5.067 mg; ISR: 0.965 mg; GA: 1.416 mg; GB: 0.680 mg; GC: 0.796 mg; BB: 0.326 mg; TSIIA: 0.624 mg; SAB: 3.84 mg). After dosing for 0 min, 5 min, 15 min, 20 min, 30 min, 45 min, 1 h, 1.5 h, 2 h, 3 h, 4 h, 6 h, 8 h, 12 h, and 24 h, blood samples (500 μL) were drawn from the retro-orbital plexus of each mouse and collected in heparinized tubes according to the specific schedule. All the collected blood samples were immediately centrifuged at 13 000 rpm for 10 min. The plasma layer was transferred to clean tubes and stored at −20 °C until analysis. The plasma concentrations of QCT, ISR, KMF, GA, GB, GC, BB, TSIIA, and SAB were expressed as the mean ± standard deviation (SD), and the mean concentration–time curve was plotted. Non-compartmental pharmacokinetic parameters were computed using DAS 2.1.1 software.

## 3. Results and discussion

### 3.1 Identification of prototype constituents and metabolites in drug-containing plasma

**Fig. 2:**
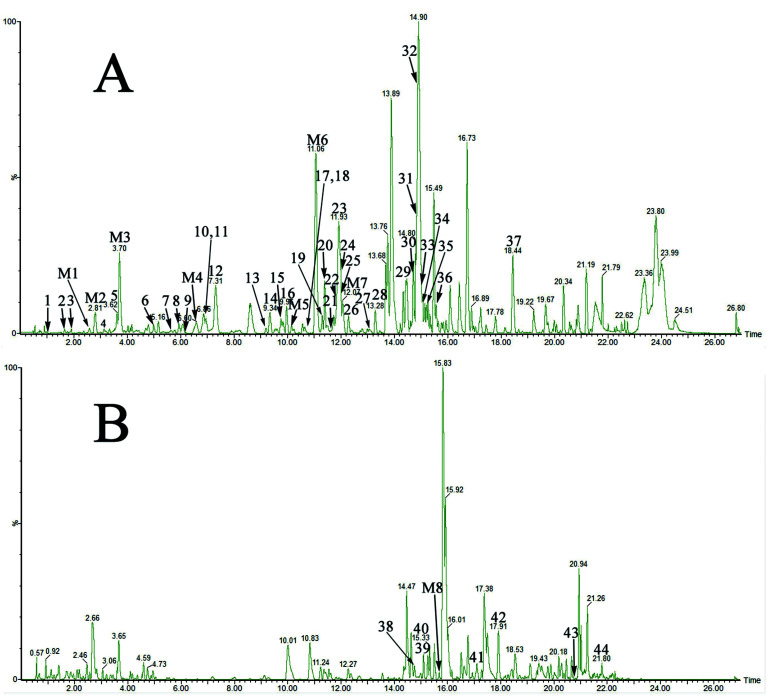
The UPLC-Q-TOF-MS results for drug-containing plasma. (A) negative ionisation mode-analysis of drug-containing plasma. (B) positive ionisation-mode analysis of drug-containing plasma.

The optimized LC-MS protocol was used for the separation and identification of compounds in drug-containing plasma. Exogenous constituents of YDXNT in the plasma were divided into prototype components and metabolites. Ultimately, a total of 52 compounds, including 44 prototype constituents and 8 metabolites, were detected by comparison to the blank plasma sample (Fig. 2, Tables 2 and 3).

(1) Chromatographic and MS data of absorbed constituents analysed by UPLC-Q-TOF-MS in negative mode. (2) Chromatographic and MS data of absorbed constituents analysed by UPLC-Q-TOF-MS in positive mode

**Table:** 

Peak no.	*RT* (min)	[M − H]^−^	Tolerance ppm	MS^2^	Molecular formula	Tentatively identity	Source
1	1.01	217.0338				Unknown	16
2	2.03	197.0449	2.284	135.0470 [M − H–C_2_H_2_O]^−^	C_9_H_10_O_5_	Tanshinol	16
3	2.37	153.0188	3.691	109.0326 [M − H–CO_2_]^−^	C_7_H_6_O_4_	Protocatechuic acid	16
4	3.18	137.0233	−0.150	108.0453	C_7_H_6_O_3_	Protocatechualdehyde	16
5	3.62	179.0349	5.668		C_9_H_8_O_4_	Caffeic acid	16
6	4.99	755.2035	6.4	301.0343 [M − H–2C_6_H_10_O_4_–C_6_H_10_O_5_]^−^	C_33_H_40_O_20_	Quercetin 3-*O*-[2-*O*,6-*O*-bis(α-l-rhamnosyl)-β-d-glucoside]	16
7	5.82	193.0501	0.0	178.9783, 134.9963	C_10_H_10_O_4_	Ferulic acid	16
8	6.00	739.2123	5.810	593.1466 [M − H–C_6_H_10_O_4_]^−^	C_33_H_40_O_19_	Kaempferol 3-*O*-[2-*O*,6-*O*-bis(α-l-rhamnosyl)-β-d-glucoside] (c)	16
				447.0882 [M − H–2C_6_H_10_O_4_]^−^			
				285.0379 [M − H–2C_6_H_10_O_4_–C_6_H_10_O_5_]^−^			
9	6.19	769.2180	0.741	315.0522 [M − H–2C_6_H_10_O_4_–C_6_H_10_O_5_]^−^	C_34_H_42_O_20_	Isorhamnetin 3-*O*-[2-O,6-*O*-bis (α-l-rhamnosyl)-β-d-glucoside]	16
10	6.73	609.1464	2.280	463.0923 [M − H–C_6_H_10_O_4_]^−^	C_27_H_30_O_16_	Quercetin 3-*O*-[6-*O*-(α-l-rhamnosyl)-β-D-glucoside]	16
				301.0345 [M − H–C_6_H_10_O_4_–C_6_H_10_O_5_]^−^			
11	6.83	325.0914	−0.9	163.1106	C_15_H_18_O_8_	Bilobalide	16
12	7.31	439.1245	2.305	411.1272 [M − H–CO]^−^	C_20_H_24_O_11_	Ginkgolide C	16
				383.1348 [M − H–2CO]^−^			
13	9.20	609.1459	2.280	477.0924 [M − H–C_6_H_10_O_5_]^−^	C_27_H_30_O_16_	Quercetin 3-*O*-[6-*O*-(α-l-rhamnosyl)-β-d-glucoside]	16
				301.0343 [M − H–C_6_H_10_O_4_–C_6_H_10_O_5_]^−^			
14	9.34	593.1501	0.006	285.0379 [M − H–C_6_H_10_O_4_–C_6_H_10_O_5_]^−^	C_27_H_30_O_15_	Kaempferol 3-*O*-[6-*O*-(α-l-rhamnosyl)-β-d-glucoside]	16
15	9.76	623.1625	2.951	315.0522 [M − H–C_6_H_10_O_4_–C_6_H_10_O_5_]^−^	C_28_H_32_O_16_	Isorhamnetin 3-*O*-[6-*O*-(α-l-rhamnosyl)-β-d-glucoside]	16
16	9.96	577.1567	2.630	269.0482 [M − H–C_6_H_10_O_4_–C_6_H_10_O_5_]^−^	C_27_H_30_O_14_	Apigenin 7-*O*-rutinoside	16
17	10.52	359.0775	3.777	161.0258 [M − H–C_9_H_10_O_5_]^−^	C_18_H_16_O_8_	Rosmarinic acid	16
18	10.57	593.1501	0.006	285.0382 [M − H–C_6_H_10_O_5_–C_6_H_10_O_4_]^−^	C_27_H_30_O_15_	Kaempferol 3-*O*-[2-*O*-(β-d-glucosyl)-α-l-rhamnoside] (a)	16
19	11.30	755.1843	3.323	609.1464 [M − H–C_6_H_10_O_4_]^−^	C_36_H_36_O_18_	Quercetin 3-*O*-[2-*O*-(6-*O*-β-hydroxy-*trans*-cinnamoyl)-β-d-glucosyl)-*α*-l-rhamnoside]	16
				301.0343 [M − H–2C_6_H_10_O_4_–C_6_H_10_O_5_]^−^			
20	11.39	977.5346 [M + HCOO^−^]^−^	3.099	931.5281 [M − H]^−^	C_47_H_81_O_18_	Panaxnotoginosides R1	16
				799.4871 [M − H–C_5_H_8_O_4_]^−^	C_48_H_82_O_20_		
				637.4261 [M − H–C_5_H_8_O_4_–C_6_H_10_O_5_]^−^			
				475.3802 [M − H–C_5_H_8_O_4_–2C_6_H_10_O_5_]^−^			
21	11.67	717.1407	−0.6011	519.0934 [M − H–C_9_H_10_O_5_]^−^	C_36_H_30_O_16_	Salvianolic acid B	16
				321.0412 [M − H–2C_9_H_10_O_5_]^−^			
22	11.91	407.1346	2.312	379.1448 [M − H–CO]^−^	C_20_H_24_O_9_	Ginkgolide A	16
				351.1444 [M − H–2CO]^−^			
23	11.93	423.1321	8.335	395.1347 [M − H–CO]^−^	C_20_H_24_O_10_	Ginkgolide B	16
				367.1410 [M − H–2CO]^−^			
24	11.98	845.4844 [M + HCOO^−^]^−^	−5.810	799.4748 [M − H]^−^	C_42_H_73_O_14_	Ginsenoside Rg1	16
				637.4261 [M − H–C_6_H_10_O_5_]^−^	C_43_H_74_O_16_		
				475.3802 [M − H–2C_6_H_10_O_5_]^−^			
25	12.01	991.5478 [M + HCOO^−^]^−^	0.584	945.5412 [M − H]^−^	C_48_H_83_O_18_	Ginsenoside Re	16
				783.4870 [M − H–C_6_H_10_O_5_]^−^	C_49_H_84_O_20_		
				621.4357 [M − H–2C_6_H_10_O_5_]^−^			
				459.3839 [M − H–3C_6_H_10_O_5_]^−^			
26	12.29	739.1883	2.332	593.1501 [M − H–C_6_H_10_O_4_]^−^	C_36_H_36_O_17_	Kaempferol 3-*O*-[2-*O*-(6-*O*-β-hydroxy-*trans*-cinnamoyl)-β-d-glucosyl)-α-l-rhamnoside] (b)	16
				285.0379 [M − H–2C_6_H_10_O_4_–C_6_H_10_O_5_]^−^			
27	13.10	301.0343	0.07	273.0384 [M − H–CO]^−^	C_15_H_10_O_7_	Quercetin	16
				151.0067 [M − H–C_8_H_8_O_3_]^−^			
28	13.28	637.1726			C_29_H_34_O_16_	Unknown	16
29	14.47	269.0482	3.939	225.1175 [M − H–CO_2_]^−^	C_15_H_10_O_5_	Apigenin	16
30	14.66	285.0453	−5.138	211.0411 [M − H–2CO–H_2_O]^−^	C_15_H_10_O_6_	Kaempferol	16
31	14.83	1153.5948 [M + HCOO^−^]^−^	−4.546	1107.5925 [M − H]^−^	C_54_H_93_O_23_	Ginsenoside Rb1	16
				945.5376 [M − H–C_6_H_10_O_5_]^−^	C_55_H_94_O_25_		
				783.4909 [M − H–2C_6_H_10_O_5_]^−^			
				621.4412 [M − H–3C_6_H_10_O_5_]^−^			
				459.3687 [M − H–4C_6_H_10_O_5_]^−^			
32	14.88	315.0522	7.208	271.0235 [M − H–CO_2_]^−^	C_16_H_12_O_7_	Isorhamnetin	16
33	14.97	829.4974 [M + HCOO^−^]^−^	3.619	783.4909 [M − H]^−^	C_41_H_71_O_13_	Ginsenoside F2/20(s)-ginsenoside Rg3	16
				637.4261 [M − H–C_6_H_10_O_4_]^−^	C_42_H_72_O_15_		
				475.3802 [M − H–C_6_H_10_O_4_–C_6_H_10_O_5_]^−^			
34	15.05	683.4370 [M + HCOO^−^]^−^	0.748	637.4310 [M − H]^−^	C_36_H_62_O_9_	Ginsenoside F1/ginsenoside Rh1	16
				475.3784 [M − H–C_6_H_10_O_5_]^−^	C_37_H_63_O_11_		
35	15.22	683.4365 [M + HCOO^−^]^−^	0.016	637.4312 [M − H]^−^	C_36_H_62_O_9_	Ginsenoside F1/ginsenoside Rh1	16
				475.3791 [M − H–C_6_H_10_O_5_]^−^	C_37_H_63_O_11_		
36	15.54	991.5486 [M + HCOO^−^]^−^	1.391	945.5411 [M − H]^−^	C_48_H_83_O_18_	Ginsenoside Rd	16
				783.4902 [M − H–C_6_H_10_O_5_]^−^	C_49_H_84_O_20_		
				621.4377 [M − H–2C_6_H_10_O_5_]^−^			
				459.3886 [M − H–3C_6_H_10_O_5_]^−^			
37	18.44	313.1482	4.3	269.1556 [M − H–CO_2_]^−^	C_17_H_14_O_6_	Salvianolic acid F	16
				213.1271 [M − H–CO_2_–2CO]^−^

**Table:** 

Peak no.	*RT* (min)	[M + H]^+^	Tolerance ppm	MS^2^	Molecular formula	Tentatively identity	
38	14.72	311.1254	−9.3	293.0783 [M + H–H_2_O]^+^	C_19_H_18_O_4_	Tanshinone II B	16
				265.1241 [M + H–H_2_O–CO]^+^			
39	15.10	311.1256	−7.3	293.1178 [M + H–H_2_O]^+^	C_19_H_18_O_4_	3α-Hydroxytanshinone II A	16
				275.1057 [M + H–2H_2_O]^+^			
40	15.33	309.1118	−1.085	291.1050 [M + H–H_2_O]^+^	C_19_H_16_O_4_	Tanshinaldehyde	16
				263.1079 [M + H–H_2_O–CO]^+^			
41	17.07	311.1254	−9.3	293.1173 [M + H–H_2_O]^+^	C_19_H_18_O_4_	Przewaquinone A	16
				275.1066 [M + H–2H_2_O]^+^			
				247.1115 [M + H–2H_2_O–CO]^+^			
42	17.91	279.1017	1.179	261.0872 [M + H–H_2_O]^+^	C_18_H_14_O_3_	Dihydrotanshinone I	16
				233.0954 [M + H–H_2_O–CO]^+^			
43	20.77	297.1495	3.295	279.1382 [M + H–H_2_O]^+^	C_19_H_20_O_3_	Cryptotanshinone	16
				251.1421 [M + H–H_2_O–CO]^+^			
44	21.80	295.1323	−1.935	277.1293 [M + H–H_2_O]^+^	C_19_H_18_O_3_	Tanshinone IIA	16
				249.1198 [M + H–H_2_O–CO]^+^			

(1) Chromatographic and MS data of metabolites analysed by UPLC-Q-TOF-MS in negative mode. (2) Chromatographic and MS data of metabolites analysed by UPLC-Q-TOF-MS in positive mode

**Table:** 

Peak no.	*RT* (min)	[M − H]^−^	Tolerance ppm	MS^2^	Molecular formula	Tentatively identity	Source
M1	2.59	313.0555	−1.6	137.0233 [M − H–Glu]^−^	C_13_H_14_O_9_	Protocatechuicaldehyde glucuronide	33–35
M2	2.81	441.1423	7.168	423.1321 [M − H–H_2_O]^−^	C_20_H_26_O_11_	Hydrolysis ginkgolides B	36,37,39,40
				367.1410 [M–H_2_O–2CO]^−^			
M3	3.70	425.1468	6.061	407.1346 [M − H–H_2_O]^−^	C_20_H_26_O_10_	Hydrolysis ginkgolides A	36,37,39,40
				351.1444 [M − H–H_2_O–2CO]^−^			
M4	6.60	457.1345	0.979	439.1267 [M − H–H_2_O]^−^	C_20_H_26_O_12_	Hydrolysis ginkgolides C	36,37,39,40
				383.1348 [M − H–H_2_O–2CO]^−^			
M5	10.16	445.0768	−0.7	269.0482 [M − H–Glu]^−^	C_21_H_18_O_11_	Apigenin glucuronide	41–45
				225.1175 [M − H–CO_2_]^−^			
M6	11.06	443.1534	−1.9	369.1520 [M − H–H_2_O–2CO]^−^	C_20_H_28_O_11_	Bi-ionized ginkgolides A	36,37,39,40
				351.1444 [M − H–2H_2_O–2CO]^−^			
M7	12.07	461.1082	2.3	285.0754 [M − H–Glu]	C_21_H_18_O_12_	Kaempferol-*O*-glucuronide	41–45
				211.0458 [M − H–Glu–2CO–H_2_O]

**Table:** 

Peak no.	*RT* (min)	[M + H]^+^	Tolerance ppm	MS^2^	Molecular formula	Tentatively identity	Source
M8	15.76	293.0816	0.7	278.1023 [M + H–CH_3_]	C_18_H_12_O_4_	1,2; 1,3; 2,3-dehydrotanshinone IIA	35,46–48
				275.1273 [M + H–H_2_O]^−^			
				247.1256 [M + H–H_2_O–2CO]^−^			

### 3.2 Analysis of prototype constituents of YDXNT in rat plasma

Owning to the high sensitivity of UPLC-Q-TOF-MS and extracted ion chromatograms, 44 prototype constituents were tentatively identified by comparison with standards, mass data, fragment information, and our previous compound research.^16^ Of the 44 peaks, 36 were detected in negative-ionization mode, and 7 peaks were detected in positive-ionization mode. The peaks included 15 flavonoids, 3 ginkgolide, 8 phenolic, 8 ginsenosides, and 7 diterpenoid tanshinones. Among them, 14 peaks were further confirmed with standards by comparing retention time and MS data, including peaks 10, 11, 12, 20, 21, 22, 23, 24, 26, 27, 30, 32, 36, 43, and 44.

### 3.3 Analysis of the metabolites of YDXNT in rat plasma

The full-scan mass spectrum was achieved from the plasma sample after YDXNT was orally administered to rats and compared to the blank plasma sample to discover possible metabolites in rat plasma. Eight metabolites, including 1 monophenol, 4 ginkgolides, 2 flavonoids, and 1 diterpenoid tanshinone, were tentatively identified from the plasma by comparing their retention times, deprotonated molecules, and characteristic fragments to those reported in the literature.^31–47^ The precursors of these metabolites were deduced to be protocatechuic aldehyde, ginkgolide A, ginkgolide B, ginkgolide C, apigenin, kaempferol, and tanshinone IIA. Characterization of the structures of these metabolites is described below.

**Fig. 3:**
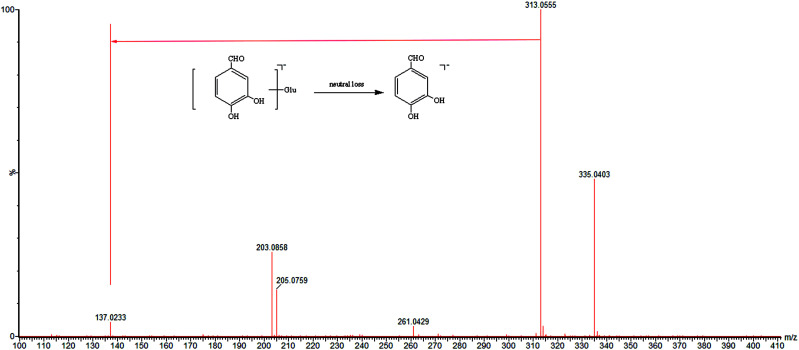
The mass spectra of protocatechuic aldehyde glucuronide in negative mode and the proposed fragmentation pathway.

In our research, one monophenolic-related metabolite (M1) was detected. M1 (*t*_R_ = 2.59 min) showed a deprotonated molecular ion at *m*/*z* 313.0555, and product ions of *m*/*z* 137.0233 were obtained. The neutral loss of 176 Da (C_6_H_8_O_6_) indicated that M1 was the glucuronide conjugate^32,33^ (Fig. 3). Based on retention time, accurate quasi-molecular ion, and MS/MS data, as well as the related literature,^32–34^ M1 was tentatively identified as the characteristic ion of protocatechuic aldehyde glucuronide.

**Fig. 4:**
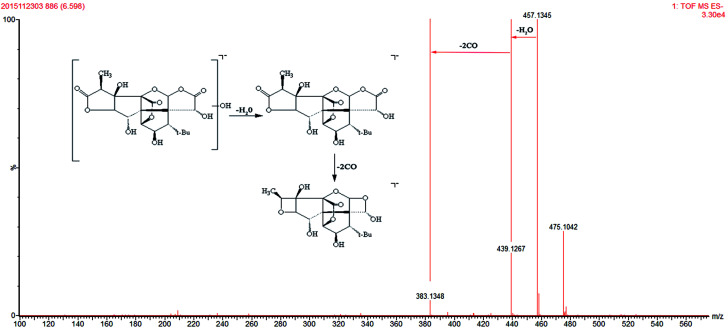
The mass spectra of hydrolysis ginkgolides C in negative mode and the proposed fragmentation pathway.

Ginkgolide-related metabolites were the main possible xenobiotic metabolites in plasma.^35,36^ In the present study, 3 ginkgolides and 4 ginkgolide-related metabolites were detected. The 3 ginkgolides (ginkgolide A, ginkgolide C and ginkgolide B) were identified by comparison to standards. The characteristic ions at *m*/*z* 351, *m*/*z* 383 and *m*/*z* 367 were observed in ginkgolide A, ginkgolide C, and ginkgolide B, respectively. The common fragmentation pattern of ginkgolide was the successive loss of 2CO.^37^ M2, M3 and M4 have [M − H]^−^ ions at *m*/*z* 441.1423, *m*/*z* 425.1468, and *m*/*z* 457.1345, which were 18 Da heavier than ginkgolide B, ginkgolide A, and ginkgolide C, respectively. The main fragment ions of M2, M3, and M4 in the MS/MS spectra were 367.1410, 351.1444, and 383.1348 by the loss of 74 Da. Three metabolites both exhibited fragment ions of [M − H–H_2_O–2CO], which have a similar mass schizolysis rule as seen for ginkgolide. According to the above analysis, we tentatively presumed that M2 is a hydrolyzed metabolite of ginkgolide B, M3 is a hydrolyzed metabolite of ginkgolide A, and M4 is a hydrolyzed metabolite of ginkgolide C (Fig. 4).^35,36,38,39^ Furthermore, M6 (*m*/*z* 443.1534) was tentatively identified as another hydrolyzed metabolite of ginkgolide A (bi-ionized ginkgolide A) by comparing deprotonated molecular [M − H]^−^ and characteristic fragments [M − H-74] to those reported in the literature.^36^

**Fig. 5:**
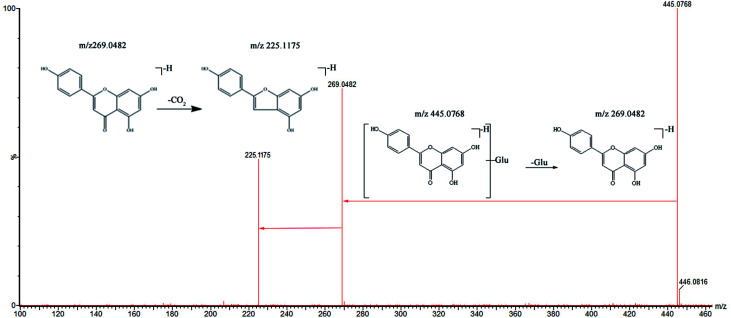
The mass spectra of apigenin glucuronide in negative mode and the proposed fragmentation pathway.

Two flavonoid-related metabolites were detected. M5 (*t*_R_ = 10.16) and M7 (*t*_R_ = 12.05) showed quasi-molecular ions at *m*/*z* 445.0768 and *m*/*z* 461.1082, respectively, in the MS spectra. Two metabolites showed product ions at *m*/*z* 269.0482 and *m*/*z* 285.0754 in the MS/MS spectra. The loss of 176 Da indicated that M5 and M7 were glucuronide conjugates. Alternatively, loss of CO_2_ from the product ion (*m*/*z* 269.0482) [M − H-176-44]^−^ and loss of 2CO and H_2_O from the product ion (*m*/*z* 285.0754) [M − H-176-74]^−^ occurred. The pathway of further fragmentation was similar to that of apigenin and kaempferol;^16^ therefore, M5 and M7 were tentatively identified as apigenin-*O*-glucuronide (Fig. 5) and kaempferol-*O*-glucuronide by comparing retention times, accurate quasi-molecular ions, and MS/MS fragmentation data as well as by referring to the related literature.^40–44^

**Fig. 6:**
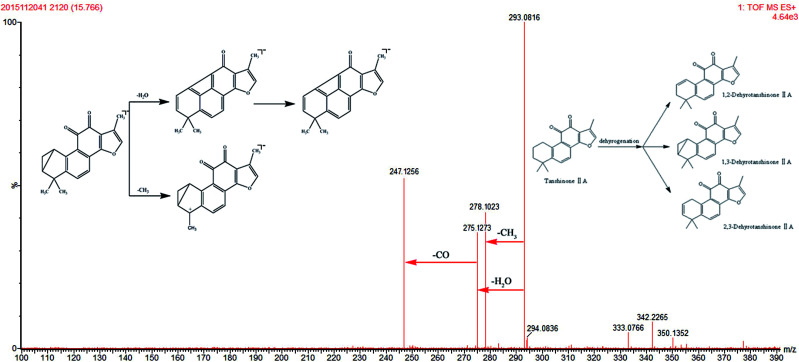
The mass spectra of 1,2; 1,3; 2,3-dehydrotanshinone IIA in positive mode and the proposed fragmentation pathway.

One tanshinone-related metabolite was tentatively identified from rat plasma on the basis of accurate deprotonated molecular data, MS/MS fragmentation data, and previous reports.^45–47^ M7 gives rise to a protonated molecule [M + H]^+^ at *m*/*z* 293.0816. This molecule was 2 Da smaller than TSIIA. The neutral loss of *m*/*z* 15, *m*/*z* 18, and *m*/*z* 28 indicated the loss of CH_3_, H_2_O, and CO, respectively (Fig. 6). Corresponding to the deprotonated molecular ion and its fragments, M7 was tentatively identified as dehydrogenated TSIIA, which has been reported in the literature.^34,45–47^

### 3.4 Assay validation

#### 3.4.1 Specificity

Under the UPLC-MS/MS conditions, a specific voltage at the optimal value was set to obtain the best sensitivity and specificity for each analyte. According to the results, there was no obvious interference from endogenous components, indicating good method selectivity. The chromatographic peaks were at retention times of 2.435, 2.580, 2.557, 2.499, 2.494, 2.324, 2.454, 3.384, 2.285 and 2.612 min for QCT, ISR, KMF, GA, GB, GC, BB, TSIIA, SAB and IS, respectively.

#### 3.4.2 Linearity

The calibration curves showed good linearity (*R*^2^ > 0.9931) over the concentration ranges of the nine constituents in rat plasma. The correlation coefficients of the standard curves and the linear ranges of the plasma are listed in Table 4.

**Table:** Regression data and LLOQs of BB, GA, GB, GC, QCT, KMF, and ISR

Biosamples	Analyte	Linear range (ng mL^−1^)	Linear equation	*R* ^2^	LLOQ (ng mL^−1^)
Plasma	QCT	1–100	*Y* = 0.0443*X* + 0.0621	0.9993	1
	ISR	1–100	*Y* = 1.8431*X* − 16.561	0.9982	1
	KMF	1–100	*Y* = 0.0031*X* + 0.0222	0.9971	1
	GA	1–100	*Y* = 0.00741*X* + 0.3012	0.9987	1
	GB	1–100	*Y* = 0.1355*X* + 1.3265	0.9987	1
	GC	1–100	*Y* = 0.0541*X* + 0.4030	0.9995	1
	BB	1–100	*Y* = 0.0204*X* + 0.1212	0.9978	1
	TSIIA	1–100	*Y* = 0.3066*X* + 0.3920	0.9992	1
	SAB	5–500	*Y* = 0.0063*X* + 0.0542	0.9985	5

#### 3.4.3 Precision and accuracy

The precision and accuracy data for six replicates of the QC samples at three concentrations are shown in Table 5. The results demonstrated that the precision (RSD%) of the method was 10.26%.

**Table:** Precision and accuracy for bioactive compounds in rat plasma and brain homogenates (*n* = 18, 6 replicates per day for 3 days)

Biosamples	Analytes	Analyte concentration (ng mL^−1^)	Inter-day		Intra-day	
			Accuracy (%)	RSD (%)	Accuracy (%)	RSD (%)
Plasma	QCT	1	94.16 ± 2.36	2.51	94.56 ± 4.05	4.28
		10	107.52 ± 4.82	4.48	101.12 ± 5.32	5.26
		100	98.73 ± 6.29	6.37	96.18 ± 7.43	7.73
	ISR	1	97.75 ± 7.39	7.56	98.33 ± 6.71	6.82
		10	93.12 ± 5.48	5.88	95.26 ± 7.93	8.32
		100	102.48 ± 3.32	3.24	100.11 ± 5.19	5.18
	KMF	1	96.49 ± 5.19	6.71	94.20 ± 6.90	7.32
		10	103.28 ± 8.48	8.21	99.89 ± 10.15	10.16
		100	92.27 ± 4.31	4.67	96.37 ± 6.54	6.79
	GA	1	100.35 ± 6.40	6.38	101.18 ± 9.64	9.53
		10	97.57 ± 2.45	2.51	95.47 ± 3.19	3.34
		100	103.63 ± 8.81	8.5	101.54 ± 9.30	9.16
	GB	1	95.53 ± 4.17	4.37	94.15 ± 6.99	7.42
		10	101.13 ± 3.23	3.19	98.24 ± 5.59	5.69
		100	99.28 ± 7.45	9.87	97.11 ± 8.97	9.24
	GC	1	94.25 ± 8.31	7.5	95.35 ± 7.80	8.18
		10	101.37 ± 9.22	9.1	99.40 ± 10.20	10.26
		100	97.46 ± 5.34	5.48	95.27 ± 6.24	6.55
	BB	1	105.38 ± 7.24	6.87	101.33 ± 7.20	7.11
		10	94.21 ± 4.22	4.48	93.62 ± 5.27	5.63
		100	96.48 ± 5.29	5.48	94.71 ± 6.03	6.37
	TSIIA	1	97.36 ± 6.53	6.71	94.41 ± 4.23	3.85
		10	102.42 ± 4.73	4.62	98.56 ± 6.73	2.73
		100	106.26 ± 10.49	9.87	99.18 ± 5.59	8.93
	SAB	5	92.65 ± 6.78	7.32	103.11 ± 6.94	4.97
		50	94.57 ± 4.69	4.96	95.44 ± 7.26	6.20
		500	101.77 ± 7.82	7.68	98.84 ± 5.38	9.44

#### 3.4.4 Extraction recovery rates and matrix effects

The extraction recovery and matrix effects of each QC concentration are listed in Table 6. The extraction recoveries ranged from 76.86% to 95.77% at different concentrations in plasma. All ratios for matrix effects were in the range of 94.56–103.25% in plasma samples. Thus, no significant matrix effects were observed for the analytes.

**Table:** Extraction recovery and matrix effect for the analytes or bioactive compounds in rat plasma and brain homogenates (*n* = 6)

Biosamples	Analytes	Analyte concentration (ng mL^−1^)	Matrix effect		Extraction recovery	
			Accuracy (%)	RSD (%)	Accuracy (%)	RSD (%)
Plasma	QCT	1	100.69 ± 9.02	8.96	84.42 ± 8.51	10.08
		10	97.51 ± 3.91	4.51	79.87 ± 6.59	9.26
		100	96.51 ± 5.94	6.16	93.89 ± 3.40	3.62
	ISR	1	97.56 ± 4.78	4.9	83.03 ± 4.57	5.5
		10	96.33 ± 8.08	8.38	79.84 ± 2.27	2.85
		100	102.33 ± 10.89	10.64	90.31 ± 6.74	7.46
	KMF	1	96.13 ± 5.84	6.08	79.73 ± 5.88	7.37
		10	100.04 ± 8.39	8.38	89.14 ± 3.13	3.52
		100	98.81 ± 5.84	5.91	95.77 ± 5.63	5.88
	GA	1	103.25 ± 7.22	6.99	83.17 ± 3.24	3.89
		10	102.75 ± 8.69	8.45	87.72 ± 3.75	4.27
		100	98.48 ± 6.99	7.09	93.89 ± 3.40	3.62
	GB	1	99.29 ± 5.99	6.04	87.26 ± 3.27	5.26
		10	100.39 ± 5.82	5.8	85.72 ± 3.28	3.83
		100	94.71 ± 3.26	3.44	91.12 ± 5.14	5.64
	GC	1	102.91 ± 11.93	11.59	80.17 ± 2.12	2.64
		10	101.18 ± 9.23	9.13	87.72 ± 3.75	4.27
		100	98.50 ± 4.62	4.69	83.47 ± 6.25	7.49
	BB	1	94.25 ± 7.36	7.8	76.86 ± 5.23	6.8
		10	97.57 ± 5.82	5.97	81.33 ± 4.89	4.27
		100	94.56 ± 3.50	3.7	91.22 ± 3.18	6.01
	TSIIA	1	98.63 ± 5.99	6.07	82.84 ± 4.63	5.59
		10	94.63 ± 3.91	4.13	87.72 ± 3.75	4.27
		100	93.28 ± 5.21	5.59	93.92 ± 8.18	8.71
	SAB	5	99.11 ± 12.27	12.38	72.43 ± 6.26	8.64
		50	96.92 ± 5.83	6.02	77.65 ± 5.72	7.37
		500	103.64 ± 11.20	10.80	83.97 ± 7.24	8.62

#### 3.4.5 Stability

The data for freeze–thaw stability, short-term temperature stability, long-term stability, and autosampler stability under different storage conditions are summarized in Table 7. The results were well within the acceptable limits, therefore validating the established method for sample extraction, storage and intermittent analysis and indicating its suitability for pharmacokinetic study.

**Table:** Stability of the analytes or bioactive compounds in rat plasma and brain homogenates (*n* = 6)

Biosamples	Analytes	Analyte concentration (ng mL^−1^)	Short-term stability		Autosampler stability		Freeze–thaw strability (three cycles)		Long-term stability	
			Accuracy (%)	RSD (%)	Accuracy (%)	RSD (%)	Accuracy (%)	RSD (%)	Accuracy (%)	RSD (%)
Plasma	QCT	1	100.23 ± 1.69	1.6	103.73 ± 5.52	5.32	98.27 ± 6.33	6.44	97.57 ± 3.42	3.51
		10	97.45 ± 4.23	4.34	99.34 ± 6.73	6.76	100.31 ± 4.27	4.26	96.41 ± 5.79	6.01
		100	95.97 ± 2.36	2.46	94.22 ± 5.14	5.46	98.35 ± 7.84	7.97	93.17 ± 7.35	7.89
	ISR	1	99.24 ± 6.68	6.73	98.06 ± 8.29	8.45	103.15 ± 5.43	5.26	98.35 ± 6.27	6.38
		10	96.28 ± 5.89	6.12	97.72 ± 3.83	3.92	94.52 ± 3.68	3.89	96.44 ± 6.33	6.56
		100	102.18 ± 4.95	4.84	98.34 ± 7.61	7.74	99.63 ± 3.38	3.52	95.76 ± 8.18	8.54
	KMF	1	96.50 ± 6.47	6.71	99.17 ± 4.39	4.43	101.23 ± 8.76	8.65	98.70 ± 9.35	9.47
		10	101.22 ± 2.09	2.06	102.65 ± 5.28	5.14	103.21 ± 4.14	4.01	100.48 ± 7.44	7.4
		100	97.10 ± 3.69	3.8	95.96 ± 3.11	3.24	94.24 ± 6.63	7.04	95.21 ± 5.96	6.26
	GA	1	100.59 ± 6.08	6.04	97.28 ± 2.19	2.25	102.76 ± 5.39	5.25	98.92 ± 5.81	5.87
		10	102.54 ± 7.33	7.15	98.33 ± 3.46	3.52	98.42 ± 2.84	2.89	95.48 ± 10.28	10.77
		100	98.31 ± 5.29	5.39	96.83 ± 8.30	7.09	95.44 ± 7.51	7.87	92.19 ± 8.81	9.56
	GB	1	96.95 ± 2.87	2.96	101.66 ± 4.73	8.57	96.53 ± 5.48	5.68	98.45 ± 5.75	5.84
		10	98.25 ± 5.91	6.01	95.45 ± 5.97	6.25	97.82 ± 4.31	4.41	95.72 ± 6.59	3.83
		100	98.74 ± 4.06	4.11	99.11 ± 7.23	7.3	96.55 ± 5.94	4.64	97.38 ± 4.39	6.88
	GC	1	100.24 ± 4.70	3.1	98.49 ± 6.22	6.32	104.74 ± 8.47	8.09	100.07 ± 10.14	10.13
		10	104.11 ± 3.38	3.25	102.24 ± 5.11	5	100.15 ± 4.52	4.52	100.48 ± 8.62	8.58
		100	94.58 ± 5.63	5.96	91.17 ± 5.33	5.85	96.22 ± 5.73	5.96	93.97 ± 4.38	4.66
	BB	1	98.10 ± 7.83	7.98	103.47 ± 6.75	6.52	99.85 ± 6.27	6.28	99.15 ± 5.73	5.78
		10	99.78 ± 5.25	5.26	95.29 ± 2.77	2.91	98.88 ± 3.59	3.63	96.38 ± 8.63	8.95
		100	94.05 ± 2.61	2.78	97.44 ± 3.13	3.21	95.47 ± 4.92	5.15	95.54 ± 7.76	8.12
	TSIIA	1	96.50 ± 6.47	2.93	102.65 ± 5.28	4.33	100.31 ± 4.27	9.53	101.23 ± 8.76	3.47
		10	101.22 ± 2.09	3.41	95.96 ± 3.11	5.70	101.22 ± 2.09	7.26	103.21 ± 4.14	3.11
		100	95.96 ± 3.11	2.19	99.15 ± 5.73	4.92	95.97 ± 2.36	4.19	94.24 ± 6.63	3.58
	SAB	5	97.28 ± 2.19	3.74	96.38 ± 8.63	6.51	99.24 ± 6.68	3.24	102.76 ± 5.39	4.25
		50	96.44 ± 6.33	4.33	96.41 ± 5.79	9.18	98.88 ± 3.59	7.45	95.72 ± 6.59	5.67
		500	95.76 ± 8.18	8.21	93.17 ± 7.35	6.88	95.47 ± 4.92	6.17	97.38 ± 4.39	7.69

### 3.5 Pharmacokinetics studies

**Fig. 7:**
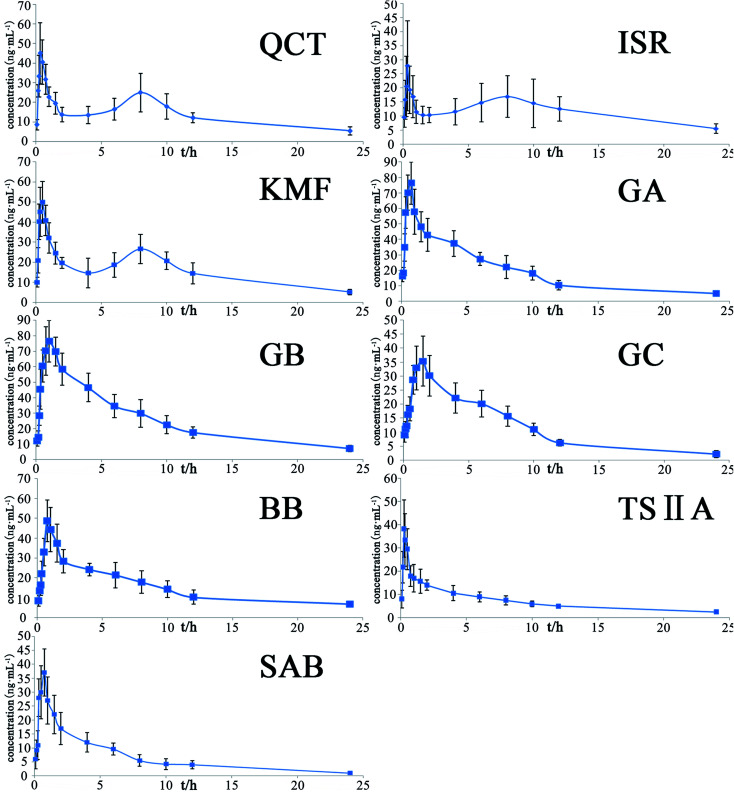
Plasma concentration–time profiles of the analytes following oral administration of the YDXNT to rats (mean ± SD, *n* = 6).

The developed and validated UPLC-QqQ-MS/MS method was successfully applied in the pharmacokinetics study of QCT, ISR, KMF, GA, GB, GC, BB, TSIIA, and SAB in rat plasma after an oral dose of 4.8 g kg^−1^ d^−1^ YDXNT. Fig. 7 shows the mean plasma concentration–time profiles of nine constituents in rat plasma. Table 8 shows the main pharmacokinetic parameters of QCT, ISR, KMF, GA, GB, GC, BB, TSIIA, and SAB in rat plasma.

**Table:** PK parameters of the seven analytes following oral administration of YXC to rats (*n* = 6)

Analytes	*C*_max_/(ng mL^−1^)	*T*_max_ (h)	AUC_0–*t*_ (ng mL^−1^ h^−1^)	AUC_0–INF_ (ng mL^−1^ h^−1^)	*T*_1/2_ (h)	MRT (h)
QCT	45.02 ± 11.28	0.33 ± 0.11	410.34 ± 73.15	412.46 ± 82.67	2.69 ± 0.65	8.44 ± 1.24
ISR	27.85 ± 8.38	0.33 ± 0.14	319.17 ± 49.27	384.49 ± 55.13	8.19 ± 2.42	13.91 ± 3.71
KMF	49.90 ± 13.82	0.50 ± 0.23	401.33 ± 84.37	539.04 ± 82.29	8.19 ± 2.42	17.11 ± 4.42
GA	76.31 ± 18.19	0.75 ± 0.29	490.92 ± 72.63	545.10 ± 78.91	7.52 ± 2.08	9.42 ± 1.77
GB	76.54 ± 15.43	1.00 ± 0.35	610.18 ± 91.45	691.00 ± 96.83	7.84 ± 3.53	10.58 ± 2.63
GC	35.35 ± 10.28	1.50 ± 0.23	281.80 ± 41.33	297.49 ± 46.59	5.45 ± 1.48	7.77 ± 2.62
BB	48.70 ± 12.34	0.75 ± 0.50	365.97 ± 48.37	383.98 ± 51.49	5.55 ± 2.67	9.62 ± 3.18
TSIIA	38.34 ± 17.35	0.25 ± 0.23	167.20 ± 69.49	182.83 ± 77.41	7.04 ± 2.18	9.89 ± 1.50
SAB	32.00 ± 15.43	0.75 ± 0.18	118.17 ± 58.46	135.72 ± 64.24	12.17 ± 3.33	9.46 ± 2.37

GA and GB have similar pharmacokinetic parameters and may be connected to similar polar constituents, similar to the absorption and elimination processes.^48^ The *C*_max_ of GC was the lowest among the four ginkgolides, indicating that GC may be partially converted to hydrolyzed ginkgolide C, as we detected. The time required to reach the maximum plasma concentration (*T*_max_) was 0.75 h for GA, 1.00 h for GB, 1.50 h for GC, and 0.75 h for BB. The *T*_1/2_ of four ginkgolides in YDXNT were 7.52 h, 7.84 h, 5.45 h, and 5.55 h. Absorption of the four ginkgolides was slower than that of the flavonols, TSIIA and SAB, while their distribution occurred relatively faster than that of the flavonols, TSIIA and SAB. Additionally, the flavonols showed double peaks in the mean plasma concentration curves. This phenomenon has been reported previously, indicating that these components might have enterohepatic recirculation.^49,50^ The double peaks suggested that enterohepatic recirculation, as well as inter-transformation among the compounds, might have occurred. One possible explanation for this phenomenon is that some of the other compounds with similar structures might have transformed into these compounds. Since drug absorption is a complex process, more detailed adsorption studies are needed to ascertain the mechanism of the double-peak phenomenon. The *C*_max_ of TSIIA (38.34 ng mL^−1^) is higher than that of SAB (32.00 ng mL^−1^), and the *T*_max_ and *T*_1/2_ of TSIIA (0.25 h, 7.04 h) are lower than those of SAB (0.75 h, 12.17 h), indicating the absorption and distribution rates of TSIIA are faster than those of SAB in rats.

## 4. Conclusion

In this research, we developed the UPLC-Q-TOF-MS method for analyzing and identifying the main absorbed constituents of YDXNT and their possible metabolites in rat plasma. A total of 52 constituents, including 44 prototype compounds and 8 metabolites, were identified or tentatively characterized. Among the prototype constituents, 15 flavonoids, 4 ginkgolides, 8 phenolic, 8 ginsenoside, and 7 diterpenoid tanshinones were identified. The metabolic routes of 8 metabolites in rat plasma after oral administration were hypothesized. The result indicated that the metabolites of YDXNT undergo the phase I metabolite pathway of hydrolysis (M2, M3, M4, and M6), dehydrogenation (M8) and the phase II metabolic route of glucuronide (M1, M5 and M7). Furthermore, a sensitive and reliable method based on UPLC-QqQ-MS was established for simultaneous quantification of nine abundant main compounds in rat plasma. The developed assay was successfully applied to assess the pharmacokinetics of ginkgetin aglycone, terpene lactones, salvianolic acid, and tanshinone from YDXNT in rat plasma. Our research clarifies for the first time the absorbed constituents, possible metabolites, and pharmacokinetics of YDXNT *in vivo*. This work provides more in-depth insight into the main active constituents of YDXNT *in vivo* as well as helpful information for clinical application. More importantly, our findings are helpful for better understanding the material foundation and action targets underlying the efficacy of YDXNT.

## Author contributions

All the authors have approved the manuscript and agree with submission to your esteemed journal.

## Conflicts of interest

There are no conflicts of interest to declare.

## Supplementary Material

RA-008-C7RA12659J-s001
