# Supplementary material for: Identification of absorbed constituents and evaluation of the pharmacokinetics of main compounds after oral administration of yindanxinnaotong by UPLC-Q-TOF-MS and UPLC-QqQ-MS
Source: RSC Adv. 2018 Apr 26;8(28):15725–39. doi: 10.1039/c7ra12659j (PMC9080097; doi:10.1039/c7ra12659j)
Supplement: RA-008-C7RA12659J-s001 [file RA-008-C7RA12659J-s001.pdf]

Electronic Supplementary Material (ESI) for RSC Advances.
This journal is © The Royal Society of Chemistry 2018

Supplement1

Optimization of UPLC-Q-TOF-MS/MS conditions

Various mobile phase systems were screened to obtain appropriate separation and better peak formation in YDXNT and its metabolites. Ultimately, acetonitrile-water was selected as the mobile phase using gradient elution since it enabled good chromatographic behavior and low background noise. Peak shapes were improved by the addition of formic acid to acetonitrile and water. To achieve better resolution, higher ionization efficiency was needed for most compounds. Both positive-ionization and negative-ionization modes were produced. In negative-ionization mode, flavonoid, ginkgolide, phenolic acid, and ginsenoside showed strong sensitivity, whereas nonpolar tanshinones were more sensitive to positive-ionization mode. Thus, both positive- and negative-ionization modes were used.

Supplement 2

Optimization of UPLC-QqQ-MS/MS conditions

Chromatographic conditions, including the reverse-phase chromatographic column, mobile phase composition, additive choice, column temperature and mobile phase flow rate, were optimized to achieve short retention times, symmetric peak shapes and satisfactory ionization. The Agilent SB-Aq C18 column was used to separate the analytes and IS with high efficiency. To achieve the desired chromatographic behavior, several mobile phases were investigated, including acetonitrile–water, methanol–water, acetonitrile–acid aqueous solution and methanol–acid aqueous solution. The acetonitrile–water system was found to have the best sensitivity. The column temperature was optimized to 30°C with a flow rate of 0.3 ml/min, and 0.1% formic acid was added to the water phase, which markedly enhanced the signal responses of all the analytes.

Under the optimized APCI conditions, the analytes and IS all exhibited higher sensitivities in negative-ionization mode than in positive-ionization mode. $[M+HCOO]^-$

ions were selected as the precursor ions for GA, and $[M-H]^-$ ions were selected as the precursor ions for other analytes. The MRM parameters, including fragmentor and collision energy, were optimized to improve the sensitivity. Other parameters such as the ESI source temperature, desolvation gas flow, nebulizer gas pressure, and capillary voltage were also optimized to improve the responses of all compounds.

Supplement 3

Method validation

The specificity of the method was evaluated by comparing the chromatograms of blank plasma obtained from six rats with those corresponding to standard plasma or the actual sample.

The linearity of the method was evaluated by analyzing nine calibration curves containing six non-zero concentrations. The calibration curves were fitted by least-square regression using $1x^{-2}$ as the weighting factor of the peak area ratio of each analyte to IS versus individual plasma concentrations. The LLOQ was determined at the lowest detectable concentration, taking into consideration a 1:10 baseline noise-calibration point ratio. Calculations were repeated six times to confirm that the precision and accuracy error was less than 20%.

The precision and accuracy were assessed by determining the concentrations of QC samples of plasma at three different concentrations on the same day and on three consecutive days. Precision was expressed as relative standard deviation (RSD), and accuracy was assessed by comparing the measured concentration with its nominal value. Calculations were repeated six times to confirm that the precision and accuracy error was less than 20%.

Extraction recovery was calculated by comparing the responses of QC samples at three levels that were spiked with analytes prior to extraction to the response of those spiked with blank plasma. The matrix effect was assessed in a similar manner. Analytes for all nine compounds were added to the extract of precipitated blank plasma to achieve three different concentrations. These peak areas were compared with those obtained by adding the same concentration of analytes in acetonitrile and were considered negligible at values below ± 15%.

The stability was tested by analyzing QC samples at three concentrations exposed to different conditions: at room temperature for 6 h before sample preparation (short-term stability), at room temperature for 12 h in the autosampler after sample preparation (autosampler stability), after three freeze-thaw cycles (-20°C to ambient temperature; freeze-thaw stability), and at -20°C for four weeks (long-term stability). The obtained results were compared to those of freshly prepared QC samples, and the percentage concentration deviation was calculated to evaluate stability.
